# A Series of Genes for Predicting Responses to Anti-Tumor Necrosis Factor α Therapy in Crohn’s Disease

**DOI:** 10.3389/fphar.2022.870796

**Published:** 2022-04-20

**Authors:** Kai Nie, Chao Zhang, Minzi Deng, Weiwei Luo, Kejia Ma, Jiahao Xu, Xing Wu, Yuanyuan Yang, Xiaoyan Wang

**Affiliations:** ^1^ Department of Gastroenterology, The Third Xiangya Hospital of Central South University, Changsha, China; ^2^ Hunan Key Laboratory of Nonresolving Inflammation and Cancer, Cancer Research Institute, Central South University, Changsha, China

**Keywords:** Crohn’s disease, antitumor necrosis factor α therapy, drug response, differentially expressed genes, hub genes

## Abstract

**Background:** Patients with Crohn’s disease (CD) experience severely reduced quality of life, particularly those who do not respond to conventional therapies. Antitumor necrosis factor (TNF)α is commonly used as first-line therapy; however, many patients remain unresponsive to this treatment, and the identification of response predictors could facilitate the improvement of therapeutic strategies.

**Methods**: We screened Gene Expression Omnibus (GEO) microarray cohorts with different anti-TNFα responses in patients with CD (discovery cohort) and explored the hub genes. The finding was confirmed in independent validation cohorts, and multiple algorithms and *in vitro* cellular models were performed to further validate the core predictor.

**Results:** We screened four discovery datasets. Differentially expressed genes between anti-TNFα responders and nonresponders were confirmed in each cohort. Gene ontology enrichment revealed that innate immunity was involved in the anti-TNFα response in patients with CD. Prediction analysis of microarrays provided the minimum misclassification of genes, and the constructed network containing the hub genes supported the core status of TLR2. Furthermore, GSEA also supports TLR2 as the core predictor. The top hub genes were then validated in the validation cohort (GSE159034; *p* < 0.05). Furthermore, ROC analyses demonstrated the significant predictive value of *TLR2* (AUC: 0.829), *TREM1* (AUC: 0.844), and *CXCR1* (AUC: 0.841). Moreover, TLR2 expression in monocytes affected the immune–epithelial inflammatory response and epithelial barrier during lipopolysaccharide-induced inflammation (*p* < 0.05).

**Conclusion:** Bioinformatics and experimental research identified TLR2, TREM1, CXCR1, FPR1, and FPR2 as promising candidates for predicting the anti-TNFα response in patients with Crohn’s disease and especially TLR2 as a core predictor.

## Introduction

Inflammatory bowel diseases (IBDs), including ulcerative colitis (UC) and Crohn’s disease (CD), are chronic intestinal inflammatory disorders. Treatment strategies for CD focus on maintaining remission and preventing recurrence. Commonly used medications to treat CD include mesalazine, locally active steroids (such as budesonide), systemic steroids, thiopurines (such as azathioprine and mercaptopurine), methotrexate, and biological therapies [such as antitumor necrosis factor α (TNFα), anti-integrin, and anti-interleukin (IL)-12/23 therapies] (Lamb et al., 2019; Torres et al., 2020). In particular, biological therapies, which include anti-TNFα antibodies (e.g., infliximab, adalimumab, and certolizumab), anti-integrin antibodies (e.g., vedolizumab and natalizumab), anti-IL-12/23 antibodies (e.g., ustekinumab), and Janus kinase (JAK) inhibitors, have been shown to be highly effective in many patients with CD. However, up to 30% of patients do not respond to initial treatment, and up to 50% of patients experience loss of response over time.

Among these therapies, anti-TNFα antibodies have been used for more than 26 years and are considered the most reliable biological therapy for the treatment of CD (van Dullemen et al., 1995; Torres et al., 2020). Loss of response to anti-TNFα therapy in patients with CD involves primary and secondary nonresponses. Primary nonresponse is defined as a failure of initial induction therapy, whereas secondary nonresponse is defined as failure after an effective period. Although inadequate drug levels and the development of immunogenicity to drug treatments contribute to some of these failures, additional heterogeneity of IBDs beyond the classical CD and UC subtypes is likely to be another vital factor (Chang, 2020). The incidence of nonresponse to anti-TNFα therapy ranges from 8% to 71% (mean: 38.5%) (Qiu et al., 2017). For infliximab, the incidence of nonresponse ranges from 11% to 71%, and the pooled incidences of nonresponse are 33% for infliximab, 30% for adalimumab, and 41% for certolizumab (Qiu et al., 2017). Many studies had explored ideal predictors for primary nonresponders in patients with irritable bowel syndrome receiving anti-TNFα therapy (Ben-Horin et al., 2014; Lopetuso et al., 2017; Gole and Potocnik, 2019). However, the incidence, causes, and predictors of primary nonresponse in patients with CD have not yet been thoroughly evaluated, and further identification of predictors of primary nonresponse in patients with CD may facilitate the identification of precision therapies and reduction of disease burden.

Accordingly, in this study, we aimed to identify novel predictors of anti-TNFα primary nonresponse in CD using independent bioinformatics analyses of multiple cohorts and experimental validation in cell models.

## Methods

### Date Screening and Selection

We searched the Gene Expression Omnibus (GEO, http://www.ncbi.nlm.nih.gov/geo/) database for Crohn’s disease data with the following inclusion criteria: 1) key words“(Crohn OR CD OR IBD OR inflammatory bowel disease) AND (anti-TNF OR infliximab OR adalimumab OR certolizumab OR golimumab)”; 2) *Homo sapiens*; 3) expression profiling by array OR high throughput sequencing; 4) submitted date <01/10/2022; and 5) datasets or series. After that, we reviewed every data under the following exclusive criteria: 1) recruited Crohn patients<6; 2) no Crohn’s anti-TNFα therapy record; 3) no anti-TNF response record; 4) responder or nonresponders <3; and 5) no clear endoscopy evaluations. For discovery cohorts, we tried to choose homogeneous data. For example, all the anti-TNFα therapy response outcomes were based on the endoscopy measure after therapy. Since adequate discovery data of candidates were lacking, we chose mixed Crohn’s disease majority’s data without a clear subtype for validation cohort’s selection in a slightly relaxed range.

### Common Differential Network Exploration

We performed differential expression analyses between responders and nonresponders to infliximab therapy using the limma R package (Team, 2013; Ritchie et al., 2015). Due to the limited recruited Crohn’s disease cohorts with the anti-TNFα response, for obtaining sufficient differential expressed genes, all significant differentially expressed genes (*p* < 0.05) were further analyzed using Gene Ontology (GO) enrichment in the Metascape database (https://metascape.org) (Zhou et al., 2019). The common differential genes in discovery cohorts were obtained by a Venn package in the UpsetR (Conway et al., 2017). Furthermore, the interactions among these genes were obtained from the STRING database (https://string-db.org/) (Szklarczyk et al., 2021). To identify the core network, we calculated the top differentially expressed genes using the degree algorithm in the CytoHubba package of Cytoscape software (version 3.8.2) (Shannon et al., 2003; Chin et al., 2014).

### Multiple Algorithms’ Confirmation and Core Predictor Exploration

We further performed prediction analysis of microarrays (PAM), a type of classification based on nearest centroids. The PAM R package provides an accurate predictor that may outperform much more complicated methods (Dabney, 2005; Korkola et al., 2009; Arijs et al., 2010). Owing to the relatively large group of nonresponsive patients in GSE16879, we selected this dataset for PAM. Genes with a minimum classification error were further analyzed and combined with the top differentially expressed genes. Interactions of the aforementioned genes were further evaluated using the degree algorithm of the CytoHubba package. Immune cell scoring in the GSE16879 dataset was calculated based on the xCell website using a curve fitting approach for linear comparison of cell types and a novel spillover compensation technique for separating them (Aran et al., 2017). Different immune cell type profiles were displayed.

### Validation in Independent Cohort and ROC Test

The top differentially expressed genes were further assessed in the discovery cohort, and the top five differentially expressed genes were further validated in the discovery cohort of responders and nonresponders with IBDs (validation cohort). Receiver operating characteristic (ROC) curve analysis was then performed to evaluate the predictive values of these top genes in response to anti-TNFα therapy in patients with CD.

### Single-Cell Portal Analysis

We explored Crohn’s disease data entitled “PREDICT 2021 paper: CD” on the single-cell portal (https://singlecell.broadinstitute.org/single_cell) held by the Broad Institute of MIT and Harvard. The Crohn’s disease single-cell data include 27 volunteers’ 201,883 single-cell transcriptomes. Then we obtained the cell types’ tSNE (t-Distributed Stochastic Neighbor Embedding) map and TLR2 expression tSNE map on the interactive visualization web tools.

### Coculture Model Proves the Value of Core Predictor

We then performed coculture of immune cells (THP1 cells, a human monocytic cell line) and colonic epithelial cells (Caco2, a human colonic epithelial cell line) to validate the bioinformatics results. Caco2 and THP1 cells were obtained from the Cancer Research Institute of Central South University, China. THP1 and Caco2 cells were cultured in RPMI-1640 or MEN medium (Gibco; Thermo Fisher United States) supplemented with 10% fetal bovine serum (Gibco; Thermo Fisher United States) at 37°C in an atmosphere containing 5% CO_2_. THP1 cells were stimulated using different concentrations of lipopolysaccharide (LPS; Sigma-Aldrich, Merck KGaA) or infliximab (Remicade; Cilag AG, Sweden), and Cell Counting Kit-8 (CCK8) assays (Dojindo, Japan) were performed to determine the appropriate concentration to use in subsequent experiments. THP1 cells were then transfected with a Toll-like receptor (TLR) 2 overexpression vector or TLR2 small interfering RNA (siRNA) using Lipo 2000 (Invitrogen, Carlsbad, CA, United States). Reverse transcription-quantitative polymerase chain reaction (RT-qPCR) and Western blotting were further performed to validate the overexpression and knockdown of TLR2 in THP1 cells (Proteintech, United States). The TLR2 inhibitor C29 (TargetMol, United States) was added to block TLR2 overexpression in the coculture system. Caco2 cells were treated with 100 ng/ml LPS for 24 h and then with 100 μg/ml infliximab for another 24 h in the lower chambers of 6-well Transwell plates. The cells were then cocultured with TLR2-overexpressing THP1 cells cultured with 50 μM C29. Total RNA from the Caco2 cells was obtained after 24 h of coculture, and epithelial inflammation and tight junctions (representing epithelial permeability) were assessed using qPCR. RT-qPCR and Western blotting were carried out as described previously (Luo et al., 2019). All primers and short hairpin sequences are listed in the supplemental data.

Statistical analysis of bioinformatics data was performed using the R package. One-way analysis of variance (ANOVA) was used to compare multiple randomized sets of data. Unpaired t-tests were used to compare double randomized data. Results with *p* values less than 0.05 were considered significant.

## Results

### Data Screening and Process

**FIGURE 1:**
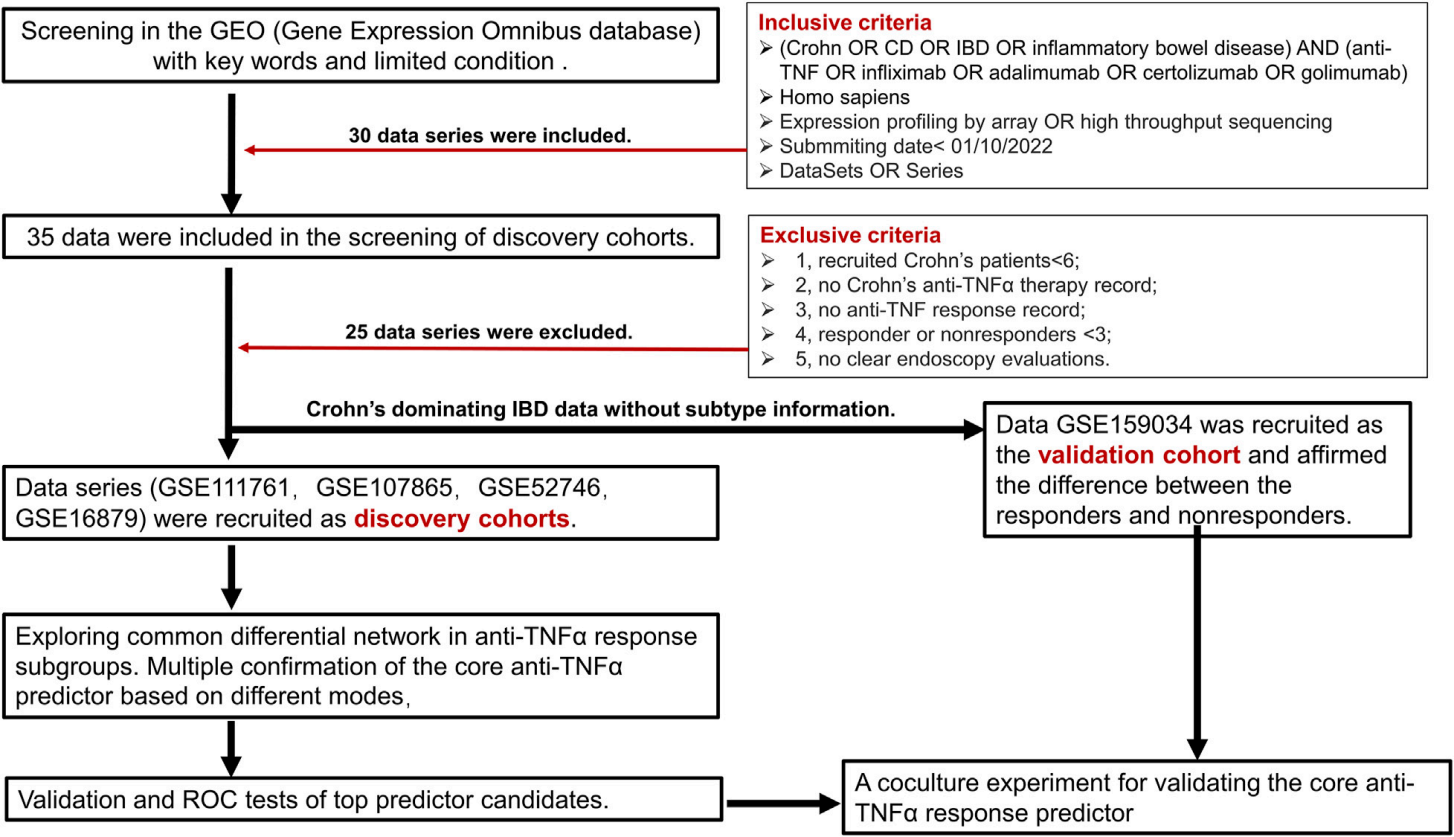
Detailed data preparation flow of the study.

A total of 30 data were included in the selection of further analysis. After carefully checking every data by the exclusion criteria, 25 data were excluded. Due to the limited eligible data for analysis, we choose total Crohn’s disease data (GSE111761, GSE107865, GSE52746, GSE16879) as discovery cohorts, which will help in revealing a reliable differential network between responders and nonresponders. For the validation cohort, we choose another mixed IBD data (GSE159034) dominated by Crohn’s disease patients without clear subtype information. The detailed information on included data is listed in Table 1. We gave priority to samples with expression profiles before the initial infliximab therapy in patients with CD (i.e., GSE16879). If there was no sampling information, we analyzed the results. First, our discovery analysis contains 47 responders and 30 nonresponders among patients with CD receiving anti-TNFα therapy. Second, differentially expressed gene networks and pathways were obtained under the p-value <0.05 for enough differential expressed genes. We performed PAM analysis and gene set enrichment analysis (GSEA) to validate the core predictor. Validation and ROC curve analyses were further performed in an independent Crohn’s disease cohort and a relatively large cohort. Coculture experimental validation was conducted. The study analysis flow is depicted in Figure 1. The four data series were downloaded from GEO and were normalized using the limma R package (Ritchie et al., 2015) (Supplementary Figure S1A).

**TABLE 1:** Detailed information of included cohorts in the combining analysis.

Reference	Platform	GEO ID	Crohn’s Ratio (%)	Responder	Nonresponder	Cohort type
Heike Schmitt et al	GPL13497	GSE111761	100	3	3	Discovery
Shai S Shen-Orr et al	GPL23159	GSE107865	100	17	5	Discovery
Azucena Salas et al	GPL17996	GSE52746	100	7	5	Discovery
Arijs et al. (2010)	GPL570	GSE16879	100	20	17	Discovery
Salvador-Martin et al. (2019)	GPL16791	GSE159034	75	6	6	Validation

### The Exploration of Core Anti-TNFα Response Differentially Expressed Genes

**FIGURE 2:**
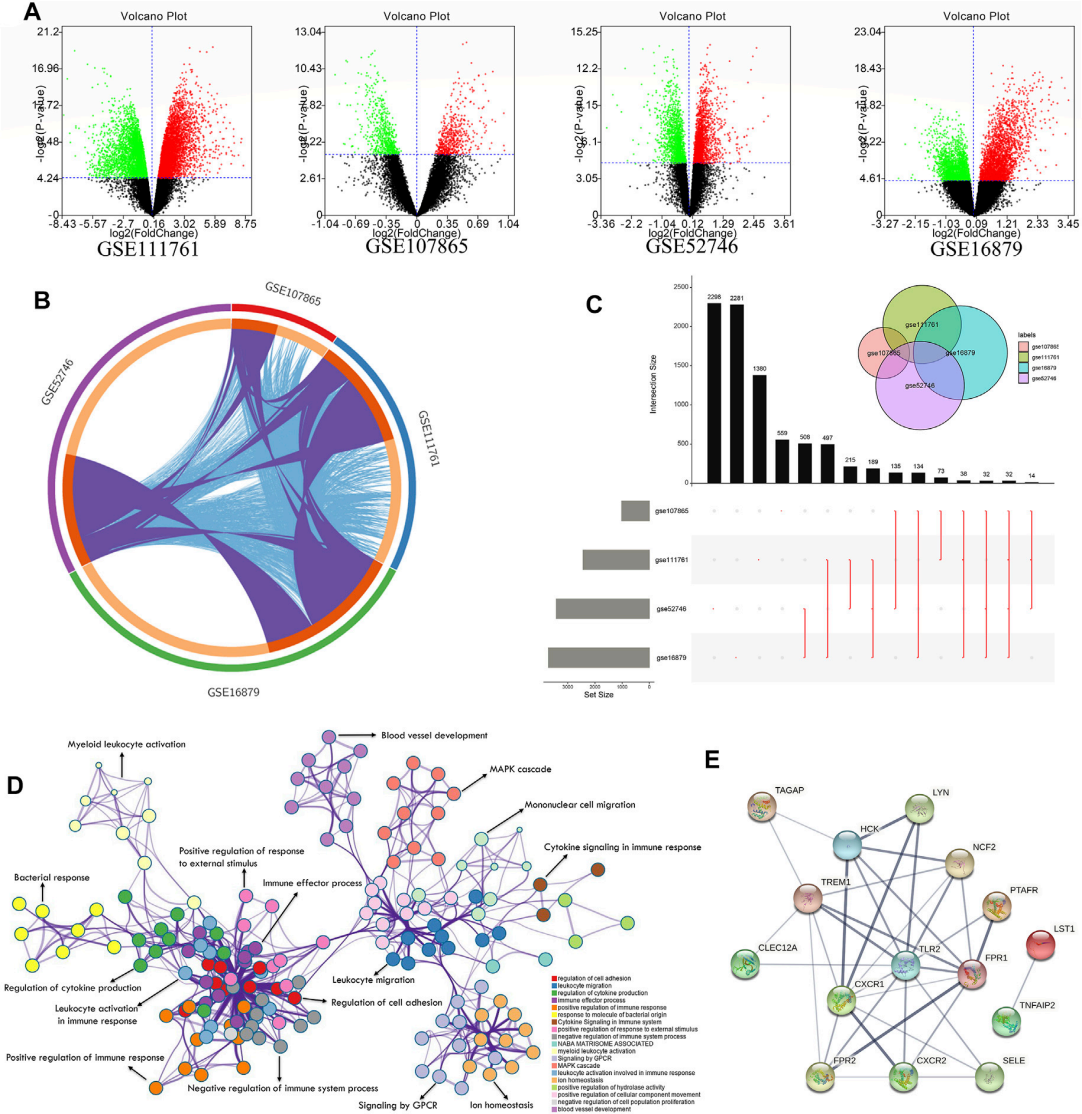
Exploration of differentially expressed gene network between anti-TNF responders and nonresponders with CD. **(A)** Volcano plot of the discovery cohorts GSE111761, GSE107865, GSE52746, and GSE16879 showing significantly upregulated genes (red dots) and downregulated genes (green dots) with *p* < 0.05 between responders and nonresponders. **(B)** Circle diagram indicating shared differentially expressed genes (purple) and interacting differentially expressed genes (blue) among the four discovery cohorts. **(C)** Plot showing shared differentially expressed genes among the four discovery cohorts. Black columns show the number of shared genes, and the gray columns show the total number of significant differentially expressed genes in each cohort. Red lines show details of the shared genes (see also Supplementary Table S1). **(D)** Network results in GO enrichment analysis. **(E)** Core network of common differentially expressed genes obtained from the STRING database.

From the analysis of differentially expressed genes between responders and nonresponders, due to the insufficient significant differential genes under the adj p-value, we identified 4690, 3422, 1020, and 2811 differentially expressed genes in GSE16879, GSE52746, GSE107865, and GSE111761 datasets, respectively, under *p* < 0.05 (Figure 2A, Supplementary Table S1). Some of the datasets shared common differentially expressed genes (purple) and interactions among genes (blue), as shown in Figure 2B. Overlaps among the four differentially expressed genes revealed that 32 common differentially expressed genes exhibited shared characteristics between responders and nonresponders of different cohorts (Figure 2C) by a Venn package in the UpsetR (Conway et al., 2017). C-X-C motif chemokine receptor (*CXCR*) 1, *CXCR2*, *TLR2*, and triggering receptor expressed on myeloid cells (*TREM1*) were common differentially expressed genes. Furthermore, GO enrichment (Zhou et al., 2019) of all differentially expressed genes showed that innate immunity was involved in the response of patients with CD to the anti-TNFα therapy. Specifically, lymphocyte activation, T-cell activation, leukocyte migration, and cell adhesion were the top GO enrichment pathways (Figure 2D, Supplementary Table S1). STRING is a database of known and predicted protein–protein interactions (Szklarczyk et al., 2021), including direct (physical) and indirect (functional) associations based on computational prediction, knowledge transfer between organisms, and interactions aggregated from other (primary) databases. Thus, interactions among common differentially expressed genes were obtained from the STRING database (Figure 2E). The degree algorithm based on this interaction network (Chin et al., 2014) revealed the core components between unresponsive and responsive subgroups. *TLR2*, *TREM1*, *CXCR1*, formyl peptide receptor 1 (*FPR1*), and *FPR2* were the top five differential genes.

### The Reinforced Validation of Core Anti-TNFα Response Feature by Independent Algorithms

**FIGURE 3:**
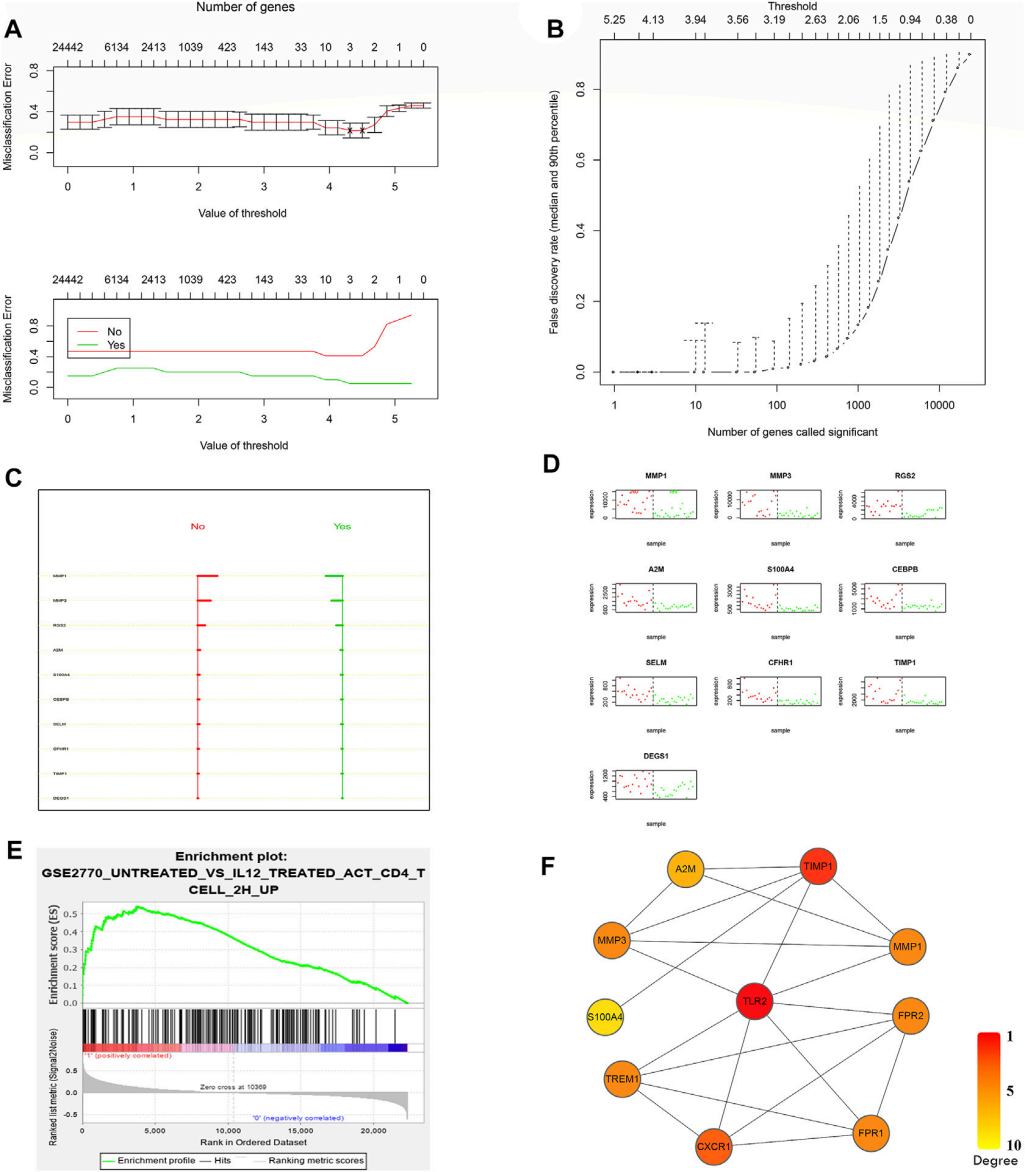
Confirmation of the core predictors after screening. **(A)** Misclassification error analysis under the PAM predictive model and model gene number. **(B)** Curve of the false discovery rate. The lowest false rate was observed when the threshold was 3.938 in the PAM model. **(C)** No-score (nonresponders) and yes-score (responders) for the best classified genes, supporting the favorable predictive value when the range widened. **(D)** Actual classification effects of these 10 risk genes. **(E)** Third-ranked GSEA results from GSE16879 nonresponders. The gene set was a Th1 immune response gene set in the database. **(F)** The core scoring network from CytoHubba for combined top differentially expressed genes and lowest misclassification genes.

To further evaluate the predictive value of the newly built model, PAM was performed based on the nearest centroid classification to classify responders and nonresponders from the GSE16879 dataset. In the PAM model, the threshold corresponding to the lowest misclassification rate was 3.938, and 10 risk genes corresponding to this threshold were considered (Figures 3A,B, Supplementary Table S2). The larger range between the no-score (nonresponders) and yes-score (responders) indicated a better value of classification (Figure 3C, Supplementary Table S2). The good classification efficiency supported the roles of these 10 genes, which included matrix metalloproteinase (*MMP*) 1, *MMP3*, regulator of G protein signaling 2, and alpha-2-macroglobulin, in our predictive model (Figure 3D, Supplementary Table S2). A combination model of the top differentially expressed genes and lowest misclassification genes was built using interaction data from the STRING database (Supplementary Figure S1C). A set of core genes, including *TLR2*, tissue inhibitor of metalloproteinases 1, *CXCR1*, and *TREM1*, emerged after the degree calculation based on the combination model in Cytoscape (Figure 3F).

GSEA is a computational method that determines whether an a priori defined set of genes shows statistically significant, concordant differences between two biological states (e.g., phenotypes) (Subramanian et al., 2005). Unlike differential analysis, which focuses on individual gene differences, GSEA derives its power by focusing on gene sets, that is, groups of genes that share common biological functions, chromosomal locations, or regulation (Subramanian et al., 2005). It includes three steps, namely, calculation of an enrichment score (ES), estimation of the significance level of the ES, and adjustment for multiple hypothesis testing (Subramanian et al., 2005). Thus, we next performed GSEA to detect the core genes between responders and nonresponders. When we treated the response to infliximab as a phenotype in the GSE16879 dataset, a Th1 immune response gene showed an enrichment score of 0.54 (*p* = 0.015), representing one of the top three enrichment gene sets (Figure 3E). Importantly, we confirmed that *TLR2* was one of the genes in the core enrichment gene list for this Th1 gene set (Supplementary Figure S1B, Supplementary Table S2). However, no other previous core genes were found in the top three core enrichment genes.

**FIGURE 4:**
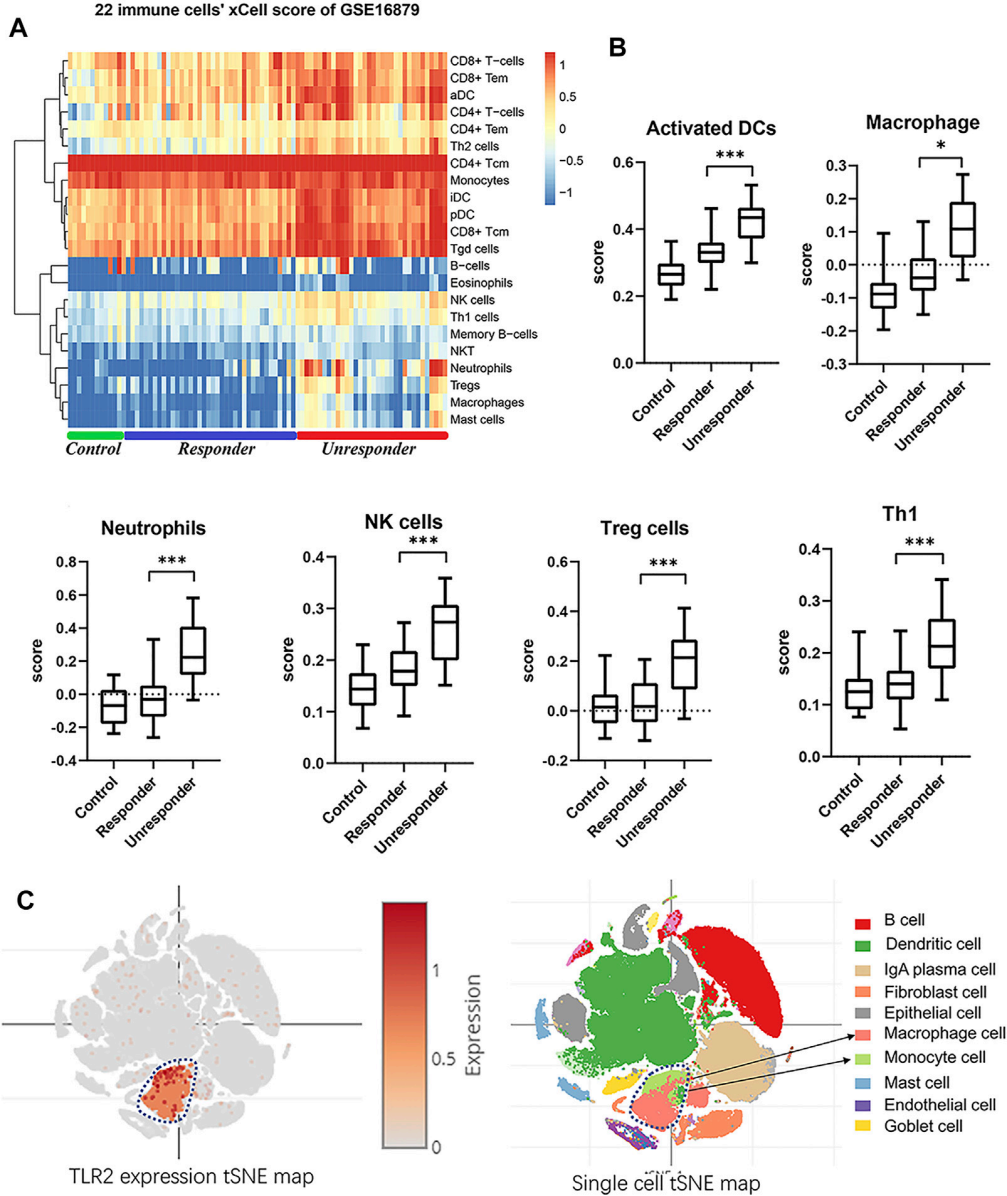
Immune scores landscape between responders and nonresponders. **(A)** Heatmap of xCell 22 immune cell scores in GSE16879. **(B)** Independent score profiles for different immune cells, including macrophages, activated dendritic cells, natural killer cells, regulatory T cells, and Th1-type cells (one-way ANOVA, *p < 0.05, ***p < 0.001). **(C)** tSNE map of TLR2 expression and cell types in Crohn’s disease single-cell sequence data.

Immune cell scoring of the GSE16879 dataset was then performed by uploading the expression data to the xCell website (Aran et al., 2017). A heatmap of immune cell scoring demonstrated divergent immune landscapes between responders and nonresponders (Figure 4A, Supplementary Table S3). The nonresponders had higher immune scores than responders and controls. Scores for macrophages, activated dendritic cells, natural killer cells, and neutrophils in all samples showed significant differences between responders and nonresponders (Figure 4B, Supplementary Table S3; one-way ANOVA, *p* < 0.05 or 0.001). Thus, we hypothesized that TLR2 in immune cells may have biological effects on the colonic response to infliximab. Moreover, after tSNE reanalysis of TLR2 expression and cell types in public single-cell sequencing data of Crohn’s disease patients. Data from the Single Cell Portal support the major contributor of *TLR2* expression in colonic tissues is the mononuclear phagocyte system, and lead to our THP-1 selection in the coculture validation (Figure 4C). Based on these findings, we chose *TLR2* as an experimental biomarker to validate the bioinformatics findings.

### The Core Predictor’s Independent Validation, ROC Tests, and Coculture Experiment

**FIGURE 5:**
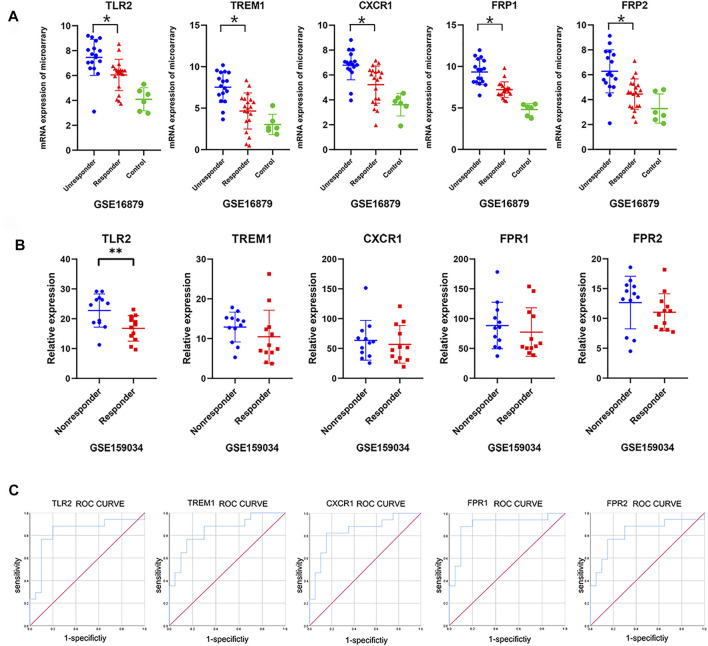
Validation of hub genes and ROC curves. **(A)** Expression profiles of the top five core genes obtained from the above network in the discovery cohort (GSE16879; one-way ANOVA, *p* < 0.05). **(B)** Validated expression profiles of the top five core genes between responders and nonresponders from an additional independent IBD cohort (GSE159034; one-way ANOVA or unpaired t-tests, *p < 0.05). **(C)** ROC curves for the top five core genes in GSE16879.

To validate and evaluate these newly built predictors’ value, we first displayed the basic expression profiles of top differential genes using a scatter plot for GSE16879 (Figure 5A). The expression signature in the discovery cohort was also validated in independent IBD cohort (GSE159034) including 9 Crohn’s disease patients. Importantly, we confirmed the significant differences in *TLR2*, and consistent difference in *TREM1*, *CXCR1*, *FPR1*, and *FPR2* between responder and nonresponder groups (Figure 5B). ROC analysis using the GSE16879 dataset also demonstrated the significant predictive value of the top five differentially expressed genes [Figure 5C; *TLR2*, area under the curve (AUC): 0.829, *p* = 0.001, 95% confidence interval (CI): 0.680–0.979; *TREM1*. AUC: 0.844, *p* < 0.001, 95% CI: 0.716–0.873; *CXCR1*, AUC: 0.841, *p* < 0.001, 95% CI: 0.708–0.974; *FPR1*, AUC: 0.894, *p* < 0.001, 95% CI: 0.778–1.0; *FPR2*, AUC: 0.824, *p* < 0.001, 95% CI: 0.678–0.969].

**FIGURE 6:**
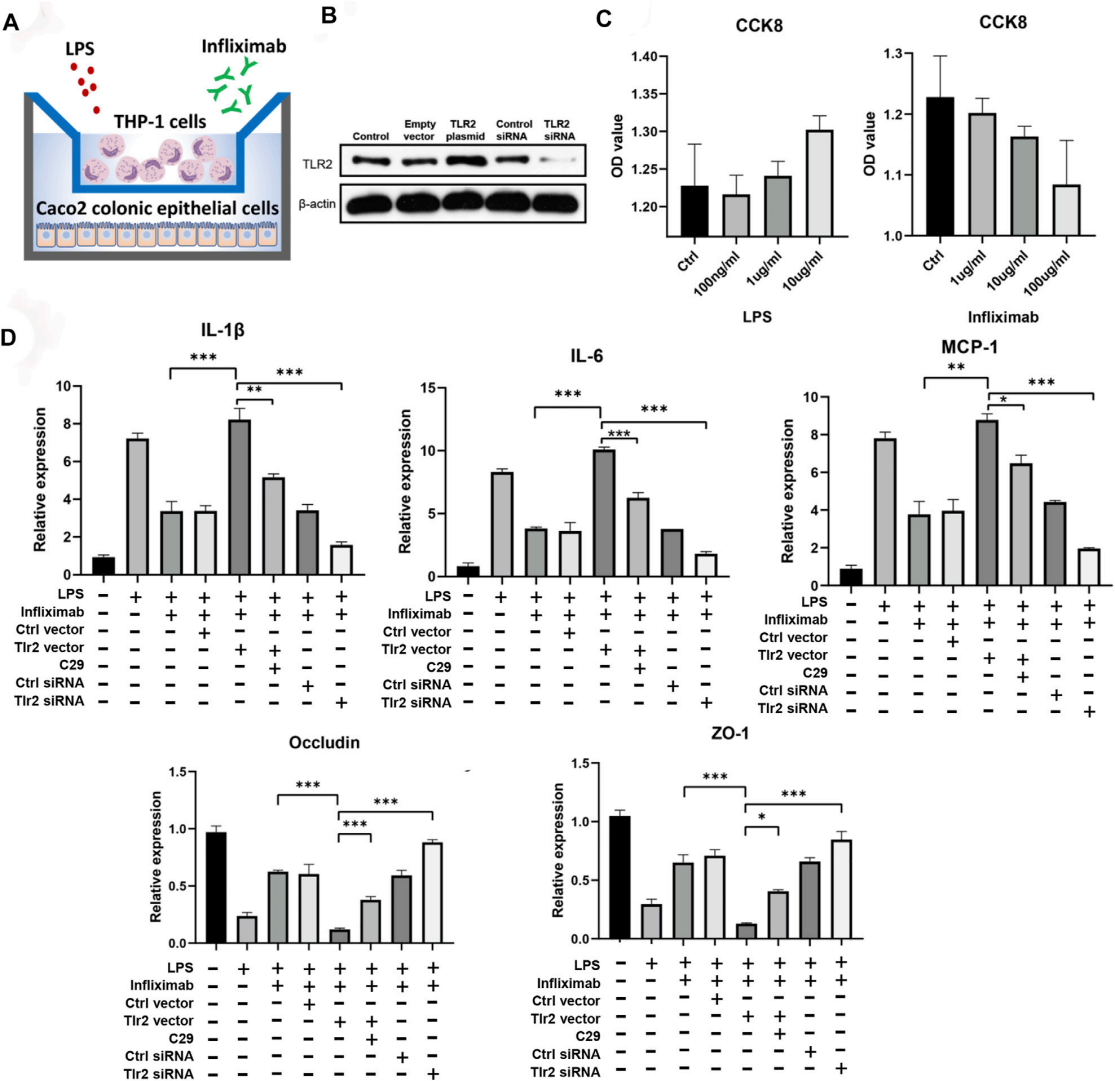
Experimental validation of core predictors. **(A)** Coculture system of THP1 and Caco2 cells. **(B)** Validated Western blotting results for TLR2 overexpression and knockdown. **(C)** CCK8 results for infliximab and LPS in Caco2 cells. **(D)** RT-qPCR results for cocultured Caco2 cells after infliximab treatment in the context of LPS-induced inflammation (one-way ANOVA, *p < 0.05, **p < 0.01, and ***p < 0.001).

A coculture system of THP1 and Caco2 cells was established to elucidate the effects of *TLR2* expression on the colonic response to anti-TNFα therapy (Figure 6A). Efficient *TLR2* knockdown was achieved in THP1 cells using siRNA (Supplementary Figure S1D), and we evaluated the effects of *TLR2* overexpression and knockdown on *TLR2* expression in THP1 cells using Western blotting (Figure 6B). CCK8 assays revealed the appropriate LPS and infliximab concentrations to use in coculture (Figure 6C). Importantly, we showed that infliximab could alleviates the amplified inflammation [i.e., *IL-1β*, *IL-*6*,* and monocyte chemotactic protein 1 (*MCP1*)] and improves the reduced epithelial permeability [i.e., occludin and zona occludens (*ZO*)-*1*] in the LPS-induced model. Furthermore, the overexpression of *TLR2* in THP1 cells amplified colonic epithelial inflammation, as measured by *IL-1β*, *IL-6*, and *MCP1* (Figure 5C; one-way ANOVA, *p* < 0.05 or 0.001), and reduced the expression of tight junction proteins, such as occludin and ZO-1, even in the context of infliximab rescue. The *TLR2* inhibitor C29 alleviated *TLR2* overexpression-induced amplification of epithelial inflammation and impaired permeability during the infliximab therapy. By contrast, *TLR2* knockdown showed an effective response to infliximab rescue after the LPS induced inflammation. (Figure 6C; one-way ANOVA, *p* < 0.05 or 0.001).

## Discussion

To date, many studies have explored the factors that predict primary response to anti-TNFα in patients with CD (Siegel and Melmed, 2009; Ben-Horin et al., 2014; Leal et al., 2015; Naviglio et al., 2018; Gisbert and Chaparro, 2020). Predictive factors include patient-related factors (age, sex, weight, smoking, and body mass index), disease-related factors (disease duration, disease location/extension, disease behavior/phenotype, disease severity, previous surgery, C-reactive protein, blood count parameters, albumin, perinuclear antineutrophil cytoplasmic antibodies, anti-*Saccharomyces cerevisiae* antibodies, fecal calprotectin, fecal lactoferrin, genetic polymorphisms, and prior anti-TNF therapy), and immune–epithelial biomarkers (several genes and protein biomarkers) (Lewis, 2011; Prieto-Perez et al., 2013; Lopetuso et al., 2017; Qiu et al., 2017; Gole and Potocnik, 2019; Gisbert and Chaparro, 2020). Mucosal genes and cytokines are also important predictors. Patients with primary nonresponse show a mixed signature, with increased *IL-1β*, *IL-17α*, *MMP3*, interferon-γ, *IL-10, IL-8*, and *S100A8* (Leal et al., 2015; Kim et al., 2021). Furthermore, the expression levels of other colonic genes, including IL-17 and IL-23, also predict the response to infliximab treatment in patients with CD (Zhang et al., 2015). Nevertheless, another study showed that anti-TNFα therapy significantly downregulates *IL-1β* and *IL-17α* in nonresponders, suggesting potential predictive value in nonresponders (Leal et al., 2015). In a study of protein biomarkers, excellent long-term (3–5 years) use of infliximab was predicted according to the dose of L-selectin used in patients (Bravo et al., 2021). In another study, high pretreatment expression of OSM was also shown to be strongly associated with the failure of the anti-TNFα therapy in a large patient cohort. Therefore, OSM may be a potential predictor of the primary response to the anti-TNFα therapy (West et al., 2017). Although many efforts had been made to identify ideal biomarkers, no single ideal predictor has been accepted in clinical guidelines for CD (Danese et al., 2015; Lamb et al., 2019; Torres et al., 2020). Therefore, in this study, we explored the identification of new predictors to improve the quality of disease management in patients with CD.

Integrated bioinformatics information from multiple cohorts can overcome the bias of single-center data and provide more reliable conclusions. In one bioinformatics analysis of IBD nonresponders to anti-TNFα therapy, IL-6 was identified as a central node in the differential gene interaction network, and the TLR and JAK pathways were identified as essential nonresponse pathways (Yuan, et al., 2017). Another multicohort bioinformatics study analyzed mixed patients with IBD who showed nonresponse; nine hub genes (*TLR4*, *TLR2*, *TLR1*, *TLR8*, *CCR1*, *CD86*, *CCL4*, *HCK*, and *FCGR2A*) were identified, and the pathway enrichment highlighted the interaction between the TLR pathway and FcγR signaling. Genes such as *TLR4*, *TLR8*, and *CCL4* have also shown predictive value in nonresponsive intestinal tissue (Liu et al., 2020). Although these findings provide meaningful information and identified several candidates for further screening, CD exhibits significant heterogeneity compared with UC, and many of these studies did not conduct independent analyses in patients with CD and did not report experimental validation. By contrast, in this study, we combined data from multiple cohorts of patients with CD, confirmed biomedical algorithms, and performed experimental validation in a coculture cell model. Overall, our findings identified *TLR2* and *CXCR1* are important components of the nonresponse pathways described previously (Bek et al., 2016; Yuan, et al., 2017; Liu et al., 2020). Another core gene identified in this study, *TREM1*, was detected previously as a primary nonresponse biomarker in a cell-centered meta-analysis (Gaujoux et al., 2019). Additionally, we also identified *FPR1* and *FPR2*, which mediate the response of phagocytic cells to the invasion of the host by microorganisms, revealing important roles in host defense and inflammation (Zhang et al., 2020). These findings are consistent with most similar studies but established novel core predictors.

Gene polymorphisms, particularly those in the TNF receptor superfamily, nuclear factor-κB pathway, IL pathway, and *TLR2/9* family, have been shown to be linked to the response to anti-TNFα therapy in patients with CD (Bek et al., 2016; Bank et al., 2019; Salvador-Martin et al., 2019). *TLR2* variants (e.g., rs4696480, rs11938228, and rs2289318) are associated with the primary nonresponse to anti-TNFα therapy in patients with CD (Bank et al., 2014; Bank, 2015), whereas other *TLR2* variants (e.g., rs1816702 and rs3804099) are associated with the primary response (Bank et al., 2014). *TLR2* variants (e.g., rs1816702) in pediatric patients with IBD have been shown to be promising markers for predicting the anti-TNF therapy response. Furthermore, *TLR2* variants (e.g., rs4696480 and rs11938228) have been shown to be associated with the response to anti-TNF treatment in patients with psoriasis (Loft et al., 2018). Although only a few known variants can influence gene expression (i.e., *TNFα* rs1799724 and *TLR9* rs187084) (Bank, 2015), we observed a direct link between *TLR2* and nonresponders. In other autoimmune diseases, such as spondyloarthropathy and Behcet’s disease, TLR2 expression is downregulated after infliximab therapy, supporting the role of *TLR2* in the response to the anti-TNFα therapy (De Rycke et al., 2005; Keino et al., 2011). *TLR2* is a typical *TLR* that induces *NF-κB* pathway-related inflammatory signaling, thereby influencing inflammatory response outcomes (Scheeren et al., 2014; Wang et al., 2019; Meng et al., 2020). *TLR2* can affect the colonic immune and epithelial barrier function (Cario et al., 2007; Scheeren et al., 2014). Higher baseline expression of colonic *TLR2* induces severe inflammatory responses (Scheeren et al., 2014; Meng et al., 2020) and is linked to nonresponse to anti-TNFα treatment. Accordingly, *TLR2* is expected to be a good predictor of nonresponse.

In this study, GSEA identified the monocyte-associated pathway as a top enriched pathway, and TLR2 is highly related to the activation of monocytes and the inflammatory reaction (De Rycke et al., 2005; Bielinski et al., 2011). At the same time, a high immune score may be associated with a high inflammatory state or immune stress response. Martin et al. (2019) built a module called GIMATS module including macrophage, DC, and fibroblast markers to predict the IBD anti-TNFα response after single-cell sequencing. A macrophage is an important contributor to this GIMATS module. Thus, macrophages’ immune score may be important. However, they also found other cell types like innate lymphoid cells and glia cells are abundant in the responders. They also found that “good cells” like ILCs and Glia enrich in the responders, while our immune scoring of xCell did not include these “good cell” types. Moreover, we introduced the immune score aim to determine which cells to manipulate *TLR2* expression in coculture experiments, and our reanalysis of single-cell data of Crohn’s disease supports that the mononuclear phagocyte system is the major contributor of *TLR2* expression in the colon and leads to the THP-1 selection in the coculture validation. In our coculture system, higher *TLR2* expression in monocytes amplified the inflammatory response and caused a worse response to infliximab treatment. Moreover, higher levels of inflammatory cytokines and reduced tight junction protein expression were associated with nonresponse. We also found that the *TLR2* inhibitor C29 rescued the nonresponse outcome and therefore linked *TLR2* expression to the response to anti-TNFα treatment. Similarly, higher colonic *TLR2* expression results in poor survival outcomes in patients with colorectal cancer (Scheeren et al., 2014). However, a separate study identified a few nonresponders among pediatric patients with IBD showing downregulation of *TLR2*. Nevertheless, the baseline disease status in responders and nonresponders with CD is different. Responders have a higher Pediatric Crohn’s Disease Activity Index than nonresponders prior to therapy, indicating more severe inflammation and elevated *TLR2* in responders (Salvador-Martin et al., 2020). Additionally, *TLR2*-knockout mice exhibit more severe colitis than wild-type mice (Cario et al., 2007). High *TL*R2 expression may result in poor outcomes, whereas low *TLR2* expression may provide some health benefits; this phenomenon may enable the identification of the optimal cutoff value for the application of *TLR2* as a biomarker.

Interestingly, such discrepancies have also been observed in studies of the *TREM1* gene in patients with CD. Nonresponse to anti-TNFα therapy in patients with CD is associated with higher blood *TREM1* levels (Verstockt et al., 2019a), whereas low blood *TREM1* levels predict better anti-TNFα response in patients with IBD (Verstockt et al., 2019b). These findings are consistent with our findings for *TREM1*. However, another study indicated that *TREM1* was upregulated in nonresponders but that low blood *TREM1* levels may predict poor anti-TNFα outcomes (Gaujoux et al., 2019). However, in the latter study, the results for the discovery and validation cohorts were not consistent; thus, these results should be carefully scrutinized (Gaujoux et al., 2019). A published comment for this article suggested different findings and agreed that the optimal *TREM1* cutoff value should be determined in additional studies (Verstockt et al., 2019a; Gaujoux et al., 2019). We also propose that the optimal cutoff for *TLR2* expression in CD should be further evaluated in a larger prospective cohort. In addition, there are several factors supporting *TLR2* as a priority predictor of anti-TNFα response. First, we found *TLR2* and *TREM1* as core differential genes, but *TLR2* is more robust after the PAM results’ interaction. Second, TLR2 is proved to be a more robust predictor than *TREM1* among the discovery cohort. Third, previous ideal IBD anti-TNFα predictors like *TREM1* are also questioned as not a robust predictor of clinical or endoscopic outcomes following adalimumab treatment in patients with UC or CD (Verstockt et al., 2022.). Up to now, anti-TNFα response biomarkers had not been divided precisely into detailed disease groups. It is difficult to find ideal predictors that cover multiple diseases at the same time. Especially Crohn’s disease is quite different from ulcerative colitis in the immune reaction and pathophysiology. This is a study designed only for Crohn’s disease patients and supports a reliable application in Crohn’s disease management. *TLR2* will provide a candidate of predictors in the Crohn’s disease anti-TNFα therapy. We support the subgroup predictors’ classification in the management of IBD. However, we will not be surprised to see the future applicability in other autoimmune diseases like rheumatoid arthritis because predictors may well indicate the TNFα’s origin and release dynamics.

However, there were some limitations to this study as well, such as the number of patients included in the study was small. Because of the coronavirus disease 2019 pandemic and the limited number of patients taking anti-TNFα therapy, frequent loss to follow-up, and the relatively high cost of biologics in China (Kennedy et al., 2020), we failed to recruit a sufficient number of eligible nonresponders into the validation cohort in our study. We believe our findings strongly support that *TLR2* is a promising predictor for the response to anti-TNFα therapy in patients with CD. Future research should focus on determining the optimal cutoff value for *TLR2* expression in a larger cohort of patients with CD.

In conclusion, bioinformatics analysis and experimental validation showed that innate immunity played critical roles in the response of patients with CD to anti-TNFα therapy. Moreover, we identified *TLR2*, *TREM1*, *CXCR1*, *FPR1*, and *FPR2* as promising candidates for predicting response to anti-TNFα therapy in patients with CD. Our findings provided evidence that *TLR2* could be a potential predictor for the anti-TNFα nonresponse in patients with CD, which could facilitate the establishment of novel approaches to alleviate disease burden.

## Data Availability

Publicly available datasets were analyzed in this study. The datasets (GSE16879, GSE52746, GSE107865, GSE111761, GSE159034) for this study can be found in the Gene Expression Omnibus database (http://www.ncbi.nlm.nih.gov/geo/). Single-cell data of Crohn’s disease entitled “PREDICT 2021 paper: CD” is available on the single-cell portal (https://singlecell.broadinstitute.org/single_cell).

## References

[B1] Aran D. Hu Z. Butte A. J. (2017). xCell: Digitally Portraying the Tissue Cellular Heterogeneity Landscape. Genome Biol. 18 (1), 220. 10.1186/s13059-017-1349-1 29141660 PMC5688663

[B2] Arijs I. Quintens R. Van Lommel L. Van Steen K. De Hertogh G. Lemaire K. (2010). Predictive Value of Epithelial Gene Expression Profiles for Response to Infliximab in Crohn's Disease. Inflamm. Bowel Dis. 16 (12), 2090–2098. 10.1002/ibd.21301 20848504

[B3] Bank S. (2015). A Cohort of Anti-TNF Treated Danish Patients with Inflammatory Bowel Disease, Used for Identifying Genetic Markers Associated with Treatment Response. Dan Med. J. 62 (5), B5087. 26050839

[B4] Bank S. Andersen P. S. Burisch J. Pedersen N. Roug S. Galsgaard J. (2014). Associations between Functional Polymorphisms in the NFκB Signaling Pathway and Response to Anti-TNF Treatment in Danish Patients with Inflammatory Bowel Disease. Pharmacogenomics J. 14 (6), 526–534. 10.1038/tpj.2014.19 24776844

[B5] Bank S. Julsgaard M. Abed O. K. Burisch J. Broder Brodersen J. Pedersen N. K. (2019). Polymorphisms in the NFkB, TNF-Alpha, IL-1beta, and IL-18 Pathways Are Associated with Response to Anti-TNF Therapy in Danish Patients with Inflammatory Bowel Disease. Aliment. Pharmacol. Ther. 49 (7), 890–903. 10.1111/apt.15187 30811631

[B6] Bek S. Nielsen J. V. Bojesen A. B. Franke A. Bank S. Vogel U. (2016). Systematic Review: Genetic Biomarkers Associated with Anti-TNF Treatment Response in Inflammatory Bowel Diseases. Aliment. Pharmacol. Ther. 44 (6), 554–567. 10.1111/apt.13736 27417569 PMC5113857

[B7] Ben-Horin S. Kopylov U. Chowers Y. (2014). Optimizing Anti-TNF Treatments in Inflammatory Bowel Disease. Autoimmun. Rev. 13 (1), 24–30. 10.1016/j.autrev.2013.06.002 23792214

[B8] Bielinski S. J. Hall J. L. Pankow J. S. Boerwinkle E. Matijevic-Aleksic N. He M. (2011). Genetic Variants in TLR2 and TLR4 Are Associated with Markers of Monocyte Activation: the Atherosclerosis Risk in Communities MRI Study. Hum. Genet. 129 (6), 655–662. 10.1007/s00439-011-0962-4 21298446 PMC3417332

[B9] Bravo F. Macpherson J. A. Slack E. Patuto N. Cahenzli J. McCoy K. D. (2021). Prospective Validation of CD-62L (L-Selectin) as Marker of Durable Response to Infliximab Treatment in Patients with Inflammatory Bowel Disease: A 5-Year Clinical Follow-Up. Clin. Transl Gastroenterol. 12 (2), e00298. 10.14309/ctg.0000000000000298 33735154 PMC7886452

[B10] Cario E. Gerken G. Podolsky D. K. (2007). Toll-like Receptor 2 Controls Mucosal Inflammation by Regulating Epithelial Barrier Function. Gastroenterology 132 (4), 1359–1374. 10.1053/j.gastro.2007.02.056 17408640

[B11] Chang J. T. (2020). Pathophysiology of Inflammatory Bowel Diseases. N. Engl. J. Med. 383 (27), 2652–2664. 10.1056/NEJMra2002697 33382932

[B12] Chin C. H. Chen S. H. Wu H. H. Ho C. W. Ko M. T. Lin C. Y. (2014). cytoHubba: Identifying Hub Objects and Sub-networks from Complex Interactome. BMC Syst. Biol. 8 (Suppl. 4), S11. 10.1186/1752-0509-8-S4-S11 25521941 PMC4290687

[B13] Conway J. R. Lex A. Gehlenborg N. (2017). UpSetR: an R Package for the Visualization of Intersecting Sets and Their Properties. Bioinformatics 33 (18), 2938–2940. 10.1093/bioinformatics/btx364 28645171 PMC5870712

[B14] Dabney A. R. (2005). Classification of Microarrays to Nearest Centroids. Bioinformatics 21 (22), 4148–4154. 10.1093/bioinformatics/bti681 16174683

[B15] Danese S. Vuitton L. Peyrin-Biroulet L. (2015). Biologic Agents for IBD: Practical Insights. Nat. Rev. Gastroenterol. Hepatol. 12 (9), 537–545. 10.1038/nrgastro.2015.135 26284562

[B16] De Rycke L. Vandooren B. Kruithof E. De Keyser F. Veys E. M. Baeten D. (2005). Tumor Necrosis Factor Alpha Blockade Treatment Down-Modulates the Increased Systemic and Local Expression of Toll-like Receptor 2 and Toll-like Receptor 4 in Spondylarthropathy. Arthritis Rheum. 52 (7), 2146–2158. 10.1002/art.21155 15986373

[B17] Gaujoux R. Starosvetsky E. Maimon N. Vallania F. Bar-Yoseph H. Pressman S. (2019). Cell-centred Meta-Analysis Reveals Baseline Predictors of Anti-tnfα Non-response in Biopsy and Blood of Patients with IBD. Gut 68 (4), 604–614. 10.1136/gutjnl-2017-315494 29618496 PMC6580771

[B18] Gisbert J. P. Chaparro M. (2020). Predictors of Primary Response to Biologic Treatment [Anti-TNF, Vedolizumab, and Ustekinumab] in Patients with Inflammatory Bowel Disease: From Basic Science to Clinical Practice. J. Crohns Colitis 14 (5), 694–709. 10.1093/ecco-jcc/jjz195 31777929

[B19] Gole B. Potočnik U. (2019). Pre-Treatment Biomarkers of Anti-tumour Necrosis Factor Therapy Response in Crohn's Disease-A Systematic Review and Gene Ontology Analysis. Cells 8 (6), 515. 10.3390/cells8060515 PMC6628089 31141991

[B20] Keino H. Watanabe T. Taki W. Okada A. A. (2011). Effect of Infliximab on Gene Expression Profiling in Behcet's Disease. Invest. Ophthalmol. Vis. Sci. 52 (10), 7681–7686. 10.1167/iovs.11-7999 21862654

[B21] Kennedy N. A. Jones G. R. Lamb C. A. Appleby R. Arnott I. Beattie R. M. (2020). British Society of Gastroenterology Guidance for Management of Inflammatory Bowel Disease during the COVID-19 Pandemic. Gut 69 (6), 984–990. 10.1136/gutjnl-2020-321244 32303607 PMC7211081

[B22] Kim K. U. Kim J. Kim W. H. Min H. Choi C. H. (2021). Treatments of Inflammatory Bowel Disease toward Personalized Medicine. Arch. Pharm. Res. 44 (3), 293–309. 10.1007/s12272-021-01318-6 33763844

[B23] Korkola J. E. Houldsworth J. Feldman D. R. Olshen A. B. Qin L. X. Patil S. (2009). Identification and Validation of a Gene Expression Signature that Predicts Outcome in Adult Men with Germ Cell Tumors. J. Clin. Oncol. 27 (31), 5240–5247. 10.1200/JCO.2008.20.0386 19770384 PMC3651602

[B24] Lamb C. A. Kennedy N. A. Raine T. Hendy P. A. Smith P. J. Limdi J. K. (2019). British Society of Gastroenterology Consensus Guidelines on the Management of Inflammatory Bowel Disease in Adults. Gut 68 (Suppl. 3), s1–s106. 10.1136/gutjnl-2019-318484 31562236 PMC6872448

[B25] Leal R. F. Planell N. Kajekar R. Lozano J. J. Ordás I. Dotti I. (2015). Identification of Inflammatory Mediators in Patients with Crohn's Disease Unresponsive to Anti-tnfα Therapy. Gut 64 (2), 233–242. 10.1136/gutjnl-2013-306518 24700437

[B26] Lewis J. D. (2011). The Utility of Biomarkers in the Diagnosis and Therapy of Inflammatory Bowel Disease. Gastroenterology 140 (6), 1817–e2. e1812. 10.1053/j.gastro.2010.11.058 21530748 PMC3749298

[B27] Liu Y. Duan Y. Li Y. (2020). Integrated Gene Expression Profiling Analysis Reveals Probable Molecular Mechanism and Candidate Biomarker in Anti-tnfα Non-response IBD Patients. J. Inflamm. Res. 13, 81–95. 10.2147/JIR.S236262 32104045 PMC7024800

[B28] Loft N. D. Skov L. Iversen L. Gniadecki R. Dam T. N. Brandslund I. (2018). Associations between Functional Polymorphisms and Response to Biological Treatment in Danish Patients with Psoriasis. Pharmacogenomics J. 18 (3), 494–500. 10.1038/tpj.2017.31 28696418

[B29] Lopetuso L. R. Gerardi V. Papa V. Scaldaferri F. Rapaccini G. L. Gasbarrini A. (2017). Can We Predict the Efficacy of Anti-TNF-α Agents? Int. J. Mol. Sci. 18 (9), 1973. 10.3390/ijms18091973 PMC5618622 28906475

[B30] Luo W. Shen Z. Deng M. Li X. Tan B. Xiao M. (2019). Roseburia Intestinalis Supernatant Ameliorates Colitis Induced in Mice by Regulating the Immune Response. Mol. Med. Rep. 20 (2), 1007–1016. 10.3892/mmr.2019.10327 31173202 PMC6625378

[B31] Martin J. C. Chang C. Boschetti G. Ungaro R. Giri M. Grout J. A. (2019). Single-Cell Analysis of Crohn's Disease Lesions Identifies a Pathogenic Cellular Module Associated with Resistance to Anti-TNF Therapy. Cell 178 (6), 1493–e20. e1420. 10.1016/j.cell.2019.08.008 31474370 PMC7060942

[B32] Meng S. Li Y. Zang X. Jiang Z. Ning H. Li J. (2020). Effect of TLR2 on the Proliferation of Inflammation-Related Colorectal Cancer and Sporadic Colorectal Cancer. Cancer Cel Int 20, 95. 10.1186/s12935-020-01184-0 PMC7104506 32256204

[B33] Naviglio S. Giuffrida P. Stocco G. Lenti M. V. Ventura A. Corazza G. R. (2018). How to Predict Response to Anti-tumour Necrosis Factor Agents in Inflammatory Bowel Disease. Expert Rev. Gastroenterol. Hepatol. 12 (8), 797–810. 10.1080/17474124.2018.1494573 29957083

[B34] Prieto-Pérez R. Cabaleiro T. Daudén E. Abad-Santos F. (2013). Gene Polymorphisms that Can Predict Response to Anti-TNF Therapy in Patients with Psoriasis and Related Autoimmune Diseases. Pharmacogenomics J. 13 (4), 297–305. 10.1038/tpj.2012.53 23337970

[B35] Qiu Y. Chen B. L. Mao R. Zhang S. H. He Y. Zeng Z. R. (2017). Systematic Review with Meta-Analysis: Loss of Response and Requirement of Anti-tnfα Dose Intensification in Crohn's Disease. J. Gastroenterol. 52 (5), 535–554. 10.1007/s00535-017-1324-3 28275925

[B36] Ritchie M. E. Phipson B. Wu D. Hu Y. Law C. W. Shi W. (2015). Limma powers Differential Expression Analyses for RNA-Sequencing and Microarray Studies. Nucleic Acids Res. 43 (7), e47. 10.1093/nar/gkv007 25605792 PMC4402510

[B37] Salvador-Martín S. López-Cauce B. Nuñez O. Laserna-Mendieta E. J. García M. I. Lobato E. (2019). Genetic Predictors of Long-Term Response and Trough Levels of Infliximab in Crohn's Disease. Pharmacol. Res. 149, 104478. 10.1016/j.phrs.2019.104478 31605784

[B38] Salvador-Martín S. Raposo-Gutiérrez I. Navas-López V. M. Gallego-Fernández C. Moreno-Álvarez A. Solar-Boga A. (2020). Gene Signatures of Early Response to Anti-TNF Drugs in Pediatric Inflammatory Bowel Disease. Int. J. Mol. Sci. 21 (9), 3364. 10.3390/ijms21093364 PMC7247673 32397546

[B39] Scheeren F. A. Kuo A. H. van Weele L. J. Cai S. Glykofridis I. Sikandar S. S. (2014). A Cell-Intrinsic Role for TLR2-MYD88 in Intestinal and Breast Epithelia and Oncogenesis. Nat. Cel Biol 16 (12), 1238–1248. 10.1038/ncb3058 25362351

[B40] Shannon P. Markiel A. Ozier O. Baliga N. S. Wang J. T. Ramage D. (2003). Cytoscape: a Software Environment for Integrated Models of Biomolecular Interaction Networks. Genome Res. 13 (11), 2498–2504. 10.1101/gr.1239303 14597658 PMC403769

[B41] Siegel C. A. Melmed G. Y. (2009). Predicting Response to Anti-TNF Agents for the Treatment of Crohn's Disease. Therap Adv. Gastroenterol. 2 (4), 245–251. 10.1177/1756283X09336364 PMC3002521 21180547

[B42] Subramanian A. Tamayo P. Mootha V. K. Mukherjee S. Ebert B. L. Gillette M. A. (2005). Gene Set Enrichment Analysis: A Knowledge-Based Approach for Interpreting Genome-wide Expression Profiles. Proc. Natl. Acad. Sci. U S A. 102 (43), 15545–15550. 10.1073/pnas.0506580102 16199517 PMC1239896

[B43] Szklarczyk D. Gable A. L. Nastou K. C. Lyon D. Kirsch R. Pyysalo S. (2021). The STRING Database in 2021: Customizable Protein-Protein Networks, and Functional Characterization of User-Uploaded Gene/measurement Sets. Nucleic Acids Res. 49 (D1), D605–D612. 10.1093/nar/gkaa1074 33237311 PMC7779004

[B44] Team R. C. (2013). R: A Language and Environment for Statistical Computing. Vienna, Austria: R Foundation for Statistical Computing. http://www.R-project.org/.

[B45] Torres J. Bonovas S. Doherty G. Kucharzik T. Gisbert J. P. Raine T. (2020). ECCO Guidelines on Therapeutics in Crohn's Disease: Medical Treatment. J. Crohns Colitis 14 (1), 4–22. 10.1093/ecco-jcc/jjz180 31711158

[B46] van Dullemen H. M. van Deventer S. J. Hommes D. W. Bijl H. A. Jansen J. Tytgat G. N. (1995). Treatment of Crohn's Disease with Anti-tumor Necrosis Factor Chimeric Monoclonal Antibody (cA2). Gastroenterology 109 (1), 129–135. 10.1016/0016-5085(95)90277-5 7797011

[B47] Verstockt B. Verstockt S. Dehairs J. Ballet V. Blevi H. Wollants W. J. (2019b). Low TREM1 Expression in Whole Blood Predicts Anti-TNF Response in Inflammatory Bowel Disease. EBioMedicine 40, 733–742. 10.1016/j.ebiom.2019.01.027 30685385 PMC6413341

[B48] Verstockt B. Al Mahi N. Pivorunas V. Smaoui N. Guay H. Kennedy N. A. (2022). DOP81 Baseline Whole-Blood Gene Expression of TREM1 Does Not Predict Clinical or Endoscopic Outcomes Following Adalimumab Treatment in Patients with Ulcerative Colitis or Crohn's Disease in the SERENE Studies. J. Crohns Colitis 16, i124–i125. 10.1093/ecco-jcc/jjab232.120 PMC11037103 37801628

[B49] Verstockt B. Verstockt S. Blevi H. Cleynen I. de Bruyn M. Van Assche G. (2019a). TREM-1, the Ideal Predictive Biomarker for Endoscopic Healing in Anti-TNF-treated Crohn's Disease Patients? Gut 68 (8), 1531–1533. 10.1136/gutjnl-2018-316845 30007919

[B50] Wang C. Baer H. M. Gaya D. R. Nibbs R. J. B. Milling S. (2019). Can Molecular Stratification Improve the Treatment of Inflammatory Bowel Disease? Pharmacol. Res. 148, 104442. 10.1016/j.phrs.2019.104442 31491469 PMC6902263

[B51] West N. R. Hegazy A. N. Owens B. M. J. Bullers S. J. Linggi B. Buonocore S. (2017). Oncostatin M Drives Intestinal Inflammation and Predicts Response to Tumor Necrosis Factor-Neutralizing Therapy in Patients with Inflammatory Bowel Disease. Nat. Med. 23 (5), 579–589. 10.1038/nm.4307 28368383 PMC5420447

[B52] Yuan Y. Yu Z. Lingzhen H. Meng S. Zhangshuo Y. Yiqing X. (2017). Bioinformatics Analyses of Key Genes Related with the Efficacy of Infliximab Treatment in Pafients with Inflammatory Bowel Diseas. Chin. J. Exp. Surg. 34 (9), 1576–1579. 10.3760/cma.j.issn.1001-9030.2017.09.044

[B53] Zhang M. Gao J. L. Chen K. Yoshimura T. Liang W. Gong W. (2020). A Critical Role of Formyl Peptide Receptors in Host Defense against Escherichia coli. J. Immunol. 204 (9), 2464–2473. 10.4049/jimmunol.1900430 32221037 PMC8459203

[B54] Zhang X. Hu J. Suo L. Yang Z. Xu T. Zhang Y. (2015). IL-17 and IL23 Expression as a Predictor of Response to Infliximab Treatment in Crohn's Disease. Zhonghua Nei Ke Za Zhi 54 (11), 940–944. Chinese. 10.3760/cma.j.issn.0578-1426.2015.11.008 26922821

[B55] Zhou Y. Zhou B. Pache L. Chang M. Khodabakhshi A. H. Tanaseichuk O. (2019). Metascape Provides a Biologist-Oriented Resource for the Analysis of Systems-Level Datasets. Nat. Commun. 10 (1), 1523. 10.1038/s41467-019-09234-6 30944313 PMC6447622

