# Supplementary material for: A Series of Genes for Predicting Responses to Anti-Tumor Necrosis Factor α Therapy in Crohn’s Disease
Source: Front Pharmacol. 2022 Apr 20;13:870796. doi: 10.3389/fphar.2022.870796 (PMC9065476; doi:10.3389/fphar.2022.870796)
Supplement: Supplementary file 4 [file DataSheet1.DOCX]

Supplementary Material

# Supplementary Data

**Primer sequence:**

actin

F ACCCTGAAGTACCCCATCGAG

R AGCACAGCCTGGATAGCAAC

ZO-1

F TGGCACATCAGCACGATTTC

R CAAACAGACCAAGCCAGCAC

Occludin

F AGGGTCGGGCCCAGTTG

R GGCTGAGAGAGCATTGGTCG

IL6

F GCAATAACCACCCCTGACCCAA

R GCTACATTTGCCGAAGAGCC

MCP1

F CCTAGCTTTCCCCAGACACC

R AAAAGCAATTTCCCCAAGTCTC

IL1β

F CCCTCTGTCATTCGCTCCC

R TAAAGAGAGCACACCAGTCCA

**TLR2 siRNA**

**shRNA1**

GGAAGATAATGAACACCAAGATTCAAGAGATCTTGGTGTTCATTATCTTCCTTTTTT

**shRNA2**

GCTGACATCCAATGGAATTAATTCAAGAGATTAATTCCATTGGATGTCAGCTTTTTT

**shRNA3**

GCAAGTGGATCATTGACAATATTCAAGAGATATTGTCAATGATCCACTTGCTTTTTT

**TLR2 Primer sequence**

F: CGCAAATGGGCGGTAGGCGTG

R: CCTCTACAAATGTGGTATGGC

**Supplement Tables**

**Supplement Table S1.** Significant differentially expressed genes in four discovery cohorts, shared differentially expressed genes among the discovery cohorts, GO enrichment results, and interactions of 32 shared differentially expressed genes.

**Supplement Table S2.** PAM predictive results, GSEA results, and interactions of combined top differentially expressed genes and lowest misclassification genes.

**Supplement Table S3.** Immune cell scores from GSE16879.

## 2.2 Supplementary Figures


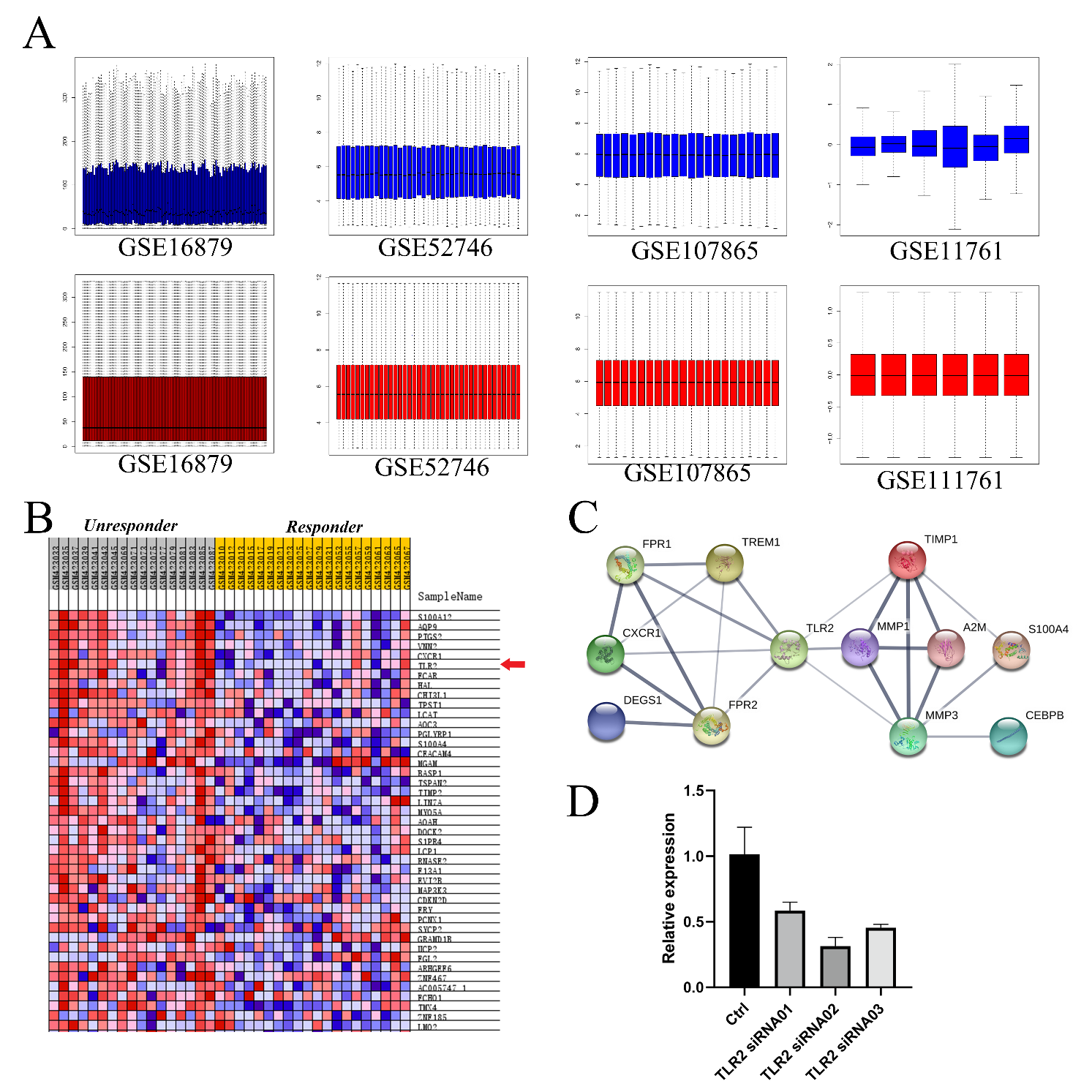


**Supplementary Figure 1.** A, Normalization results for four microarray datasets from the discovery cohorts. B, GSEA heatmaps for the core enriched genes in the third ranked Th1 response immune gene set. TLR2 is labeled in red. C, Interaction network for the combined top differentially expressed genes and lowest misclassification genes. D, RT-qPCR results for *TLR2* expression in THP1 cells transfected with TLR2 siRNA.
